# Supplementary material for: Enhancing performance with multisensory cues in a realistic target discrimination task
Source: PLoS One. 2022 Aug 5;17(8):e0272320. doi: 10.1371/journal.pone.0272320 (PMC9355224; doi:10.1371/journal.pone.0272320)
Supplement: Attachment — Submitted filename: Response to Reviewers.pdf [file pone.0272320.s001.pdf]

Dr Caterina Cinel

Essex BCI-NE Lab

School of Computer Science and Electronic Engineering

University of Essex

Wivenhoe Park, Colchester, UK

Email: ccinel@essex.ac.uk

21 April, 2022

Dear Sirs,

Many thanks for your insightful comments. We have made every effort to address them all thoroughly in our revisions. We feel that the article is now much stronger and hope that it is now acceptable for publication.

Journal Requirements:

Funding Statement: please update the funding statement as follows:

"The authors acknowledge support of the UK Defence Science and Technology Laboratory (Dstl) and Engineering and Physical Research Council (EPSRC) under grant EP/P009204/1. This is part of the collaboration between US DOD, UK MOD and UK EPSRC under the Multi-disciplinary University Research Initiative.
Dstl contributed to the early stages of the study design, and had no role in data collection and analysis, decision to publish, or preparation of the manuscript.
EPSRC had no role in study design, data collection and analysis, decision to publish, or preparation of the manuscript.
This article is an overview of UK MOD sponsored research and is released for informational purposes only. The contents of this article should not be interpreted as representing the views of the UK MOD, nor should it be assumed that they reflect any current or future UK MOD policy. The information contained in this article cannot supersede any statutory or contractual requirements or liabilities and is offered without prejudice or commitment."

We have also made several changes as requested by the Editorial Manager (after re-submssion). Those changes are described in the cover letter.

EDITOR'S COMMENTS

(-1-) theory and hypotheses

I would completely agree with R1 that your theorising on the effects of multisensory cues is a bit confusing and so are the predictions derived from your theorising. I would suggest specifying what underlying process is assumed in your situation and what process model is assumed (or could be adopted) to predict performance effects on reaction time and accuracy. At present, you

are debating general processes related to multisensory processing but it seems to fit not exactly to what you are examining empirically.

We agree that the theorising was a bit confusing in the paper. In fact, the primary purpose of the experiment, it being of an applied nature, was not to directly test specific theories, but rather to verify whether using a bimodal cue works better than using a unimodal (auditory) cue and no cues. This is now stated much more clearly in the paper.

As there are reasons to believe that an audio-visual cue could work better than an auditory-only cue based on the extensive literature in multisensory integration, we still give an overview of some of that literature in the introductory part of the article. To clarify the purposes of our paper and how it links to previous studies, we have re-structured that part of the article into two sections: "Introduction" and "Background". In the new Introduction section, we now state clearly what the context and the aims of the experiment were. In the new Background section, which is further divided into subsections for added clarity, we summarise literature relevant to different aspects of our experiment. In one of the subsections (Implications for Cueing in our Target Discrimination Task) we describe how our experiment links to that literature.
Also, part of the Abstract has been modified, and the discussion, again, to give more emphasis to the applied aspects of the paper.

(-2-) downward-sloping SOA function

More specifically, it seems you are predicting a downward-sloping foreperiod function (the variable foreperiod effect, see doi:10.1037/xhp0000561; doi:10.1016/j.actpsy.2008.08.005), which you attribute to mechanisms of inhibition of return, which is incorrect in the present situation. I suggest reconsidering your theorising in the revised version of the manuscript.

Thanks for suggesting considering the foreperiod effect. We have now added a subsection ("Temporal Preparedness") in the new Background section to describe the effect. In the Results section we then report results in relation to the foreperiod effect (lines 267-281), which include a new table (Table 4).
We also clarify that we did not expect any IOR (we never did, although, unfortunately, that was not very clear in the previous version of the manuscript), particularly because the cues in the experiment were always valid. This is now stated clearly in the background section.

(-3-) inhibition of return

The theorising on inhibition of return (IOR) is incorrect throughout the manuscript. Note that IOR is not an effect related to long intervals per se. Inhibition of return refers to the way organisms gather information from visual systems by looking. For example, in a saccadic walk during the perception of a visual scene, the information processing system tends "not" to jump back to a previous saccadic point because it would prevent the system from gathering the information efficiently. It makes no sense to confuse the foreperiod effect with IOR.

Apologies for giving the incorrect impression. As indicated above, it was never our intention to interpret our data in terms of IOR. We now have clarified that we did not expect to find IOR in our experiment, one of the main reasons being that in our experiment all trials are valid (lines 51-56).

<u>REVIEWER 1</u>

1. The explanation in 61-82 needs to be more clarified in light of the authors' experimental situation. How does multisensory cueing increase the effectiveness of cues in directing spatial attention? The authors cited some citations, such as [37], that show multisensory cues guide attention better than unisensory cues. From this, the authors seem to hypothesize that multisensory cues work better than unisensory cues in their experiments. However, the literature cited here may have examined the effects of combining unisensory cues that function on their own. If so, then there is a problem with the connection between the previous studies and this hypothesis. The reason is that the visual cue in this study did not work by itself, as this is mentioned after the results section. Additionally, the auditory cue was not very effective on its own. This point is important because it can critically affect the reason for conducting each analysis and the overall interpretation of the results. Is there a different logic from the one I described?

The reviewer is right in saying that there is a fundamental difference between the multimodal cueing experiments cited from the literature and our experiment. As he/she has pointed out, in – for example – Santangelo & Spence (2007) the two cues were each informative as unimodal cues, and when presented in combination as multisensory cues their effectiveness increased. The papers we originally cited were meant to be – as other examples in the introduction – just examples of how multisensory stimuli can be more effective at triggering a response, but not necessarily because they converge more information. However, we realise that this might have given the impression that our study and the above study are comparable in the way the cues were used (in addition to this, in our study the cues are endogenous while in the other study the cues are exogenous). As explained in our response to one of the Editor's comments (Comment 1), we have re-written and partly re-structered the introductory part of the paper, and we have added where we introduce with more clarity some of the background relevant to the experiment. Then in a separate subsection we link our experiment to the cited literature.

We have clarified the purpose of the VC cue (lines 199-204; see also response to Comment 3 below). To the analyses shown in Tables 2 and 3 we have added the VC < NC pairwise comparisons (and therefore readjusted all p-values via the Benjamini-Hochberg adjustment), with the purpose of testing the effect of the VC cue.

In addition to this, while updating Table 3 with the new p-values, we have realised that we made a mistake when typing the p-values for the RTs in the 900 SOA condition in the original version of the manuscript: this showed all non-significant p-values. The correct (and updated, after adding the VC vs. NC pairings) p-values now show significant differences between AC vs. VC, AVC vs. NC and AVC vs. VC. Apologies for this mishap.

2. In l.101, does the "task-relevant stimulus" refer only to the target or both the target and auditory cue?

The task-relevant stimulus only refers to the target. For added clarity, we have now replaced "task-relevant" with "target stimulus".

Is the auditory cue be heard from the spatially center, left, or right side of the screen? It would be better to explicitly state whether the auditory cue guides exogenous attention (e.g., a sound physically presented from the left or right or endogenous attention (e.g., a centrally presented semantic stimulus) to avoid misunderstanding by the reader.

The auditory cue was played from a loudspeaker behind the centre of the monitor, and so was 100% endogenous. This is now explicitly mentioned in the introduction and the method section.

3. Is VC the motion of the face with the lips uttering the word indicating "left" or "right" in the center of the screen as a visual cue (VC)? If my understanding is correct, this seems to be a subtle stimulus that may or may not work as a "cue." If the participants cannot instantly understand the meaning of the VC, it will not work on its own to guide attention. Is there any data that investigates this point? At least, it is necessary to describe from the subjective point of view whether the VC is a stimulus that can be understood immediately. From the description after the results, it appears that VCs do not work by themselves, but it would be easier to understand if they were mentioned in the introduction or in the methods section.

The VC condition was a non-cue condition. As clarified in the new version of the manuscript, we just called it "VC" for simplicity. In that condition a static picture of a face was presented. In the AVC condition, on the other hand, the lips in that same face moved in sync with the word being uttered (although, as explained in the new version of the manuscript the lip movement was synthetically created and was only approximate). This is now more clearly explained in the method section (lines 199-204).

4. The experimental procedure section should contain details about the stimuli to be presented. For example, how many centimeters away from the participant were the images presented, and how large were they? Also, how many visual angles between the center and the cues or targets that appeared? Without these details, the reader may be misled. In my case, I first thought that the positions of the targets presented on the left and right sides were fixed, but later I realized that there was some variation in the Results section. Note that, as mentioned in point 2, the location of the source of the auditory stimuli should also be described.

All details are now provided in the method section, where we specify the distance of participants from the screen (line 172) and give more details of the stimuli, including sizes in visual angle degrees (lines 178-186).

5. A more systematic arrangement of figures and tables throughout the Results section might be needed.

Many thanks. All tables and figures are now placed immediately following (and as close as possible) to their first reference in the text (as indicated in PlosOne manuscript template). Unfortunately, as in some cases there are a few tables cited within the same page, some of the tables have moved down quite a bit, in comparison to where they should be.

6. The Authors should provide effect sizes for statistical tests. Also, it would be easier to understand the data if variability information is added to all the graphs.

We have now added partial η2 in all ANOVA results, and in Figures 2, 3, 6 and 7 we now show the standard error of the mean.

7. In addition to the Stroop effect and the Simon effect, there is another effect that could interfere with the effect of cueing in this study: the foreperiod effect. It has been widely pointed out in the research field of the cognitive function called temporal preparation that, in an experimental situation where the target is presented after different foreperiods, the degree of readiness for the target is low at the beginning of the trial, but increases over time. The results of this study might be explained in terms of the combined effect of temporal preparation and the difference in the time it takes to process each cue. If possible, I would like to see some analysis or discussion that can shed light on this perspective. This interpretation seems to be more valid than citing examples where spatial attention is efficiently guided by the integration of multiple unisensory spatial cues which are valid on themselves. This is because neither the auditory nor visual unisensory cues did not have a major effect on the efficiency of target discrimination on their own.

Many thanks. We have now added an explanation and a detailed analysis of this effect in the article. There is a new subsection in the Background entitled “Temporal Preparedness”. The results in relation to the foreperiod effect are described in the Results section (lines 267-281), which also includes a new table (Table 4).

Best regards,

Caterina Cinel
